# How We See Black and White: The Role of Midget Ganglion Cells

**DOI:** 10.3389/fnana.2022.944762

**Published:** 2022-07-05

**Authors:** Dragos Rezeanu, Maureen Neitz, Jay Neitz

**Affiliations:** Department of Ophthalmology, University of Washington, Seattle, WA, United States

**Keywords:** black-and-white vision, color vision, computational neuroscience, retinal ganglion cell (RGC), midget ganglion cell, primate retina

## Abstract

According to classical opponent color theory, hue sensations are mediated by spectrally opponent neurons that are excited by some wavelengths of light and inhibited by others, while black-and-white sensations are mediated by spectrally non-opponent neurons that respond with the same sign to all wavelengths. However, careful consideration of the morphology and physiology of spectrally opponent L vs. M midget retinal ganglion cells (RGCs) in the primate retina indicates that they are ideally suited to mediate black-and-white sensations and poorly suited to mediate color. Here we present a computational model that demonstrates how the cortex could use unsupervised learning to efficiently separate the signals from L vs. M midget RGCs into distinct signals for black and white based only correlation of activity over time. The model also reveals why it is unlikely that these same ganglion cells could simultaneously mediate our perception of red and green, and shows how, in theory, a separate small population of midget RGCs with input from S, M, and L cones would be ideally suited to mediating hue perception.

## Introduction

Classical opponent color theory holds that the black-and-white sensations that we experience when viewing a scene are mediated by achromatic retinal ganglion cells (RGCs) that are spectrally non-opponent, responding to all wavelengths with an increase (ON cells) or a decrease (OFF cells) in firing rate (Hurvich and Jameson, 1957; DeValois et al., 1966; King-Smith and Carden, 1976). There are about a dozen functional types of RGCs if we assign each of those that come in ON and OFF pairs, such as midget and parasol RGCs, to a single functional type. However, except for the three types of RGCs with the smallest receptive fields and highest densities—the midget, parasol, and small bistratified RGCs—the rest are wide-field cells that are much too sparse and have too large of receptive fields to contribute meaningfully to the achromatic components of the highly detailed representation of the world that is inside our heads.

The midget and small bistratified RGCs are spectrally opponent, being excited by light in some areas of the visible spectrum and inhibited by others. Thus, parasol RGCs are the only candidate achromatic neurons that could be important for black-and-white pattern vision (Shapley and Hugh Perry, 1986), including reading the text on this page (Chase et al., 2003), and for being the basis for the luminance channel of human vision (Lee et al., 1988).

However, recent anatomical studies have revealed that parasol RGCs are too sparsely distributed, and they have receptive fields that are too large to support high acuity achromatic vision (Masri et al., 2020; Patterson et al., 2020). Moreover, their receptive field structure, bipolar cell circuitry (Patterson et al., 2020), projection to the magnocellular (M) pathway and dorsal stream, and response characteristics are well suited for mediating visually guided movements of our forelimbs without the intervention of visual awareness (Milner, 2012; Manookin et al., 2018; Appleby and Manookin, 2020; Liu et al., 2021), but not for transmitting information about spatial contrast and the location of edges as required for building an internal representation of the visual scene.

If we eliminate the theory that luminance, sensations of black-and-white, and achromatic pattern vision are mediated by achromatic parasol RGCs, the only possible alternative is that midget RGCs that project to the parvocellular (P) pathway are specialized to serve both high acuity black-and-white vision and color vision. The midgets are the only RGCs with high enough spatial density (making up at least 80% of RGCs in the fovea (Masri et al., 2020)) and small enough receptive fields (i.e., single cone centers) to explain human high-acuity black-and-white spatial vision. They are also the only RGCs with L vs. M spectral opponency required to explain color vision. As a result of this realization, the idea that midget RGCs must do “double-duty” mediating both high-acuity and color vision has been frequently proposed (Gouras and Zrenner, 1979; Ingling and Martinez-Uriegas, 1983; Lennie and D’Zmura, 1988; DeValois and DeValois, 1993; Lennie, 1993; Lennie and Movshon, 2005; Crook and Dacey, 2010), and except for a few holdouts, it has become the favored view among vision scientists. M/L opponent midget RGCs have a center-surround organization. This spatially opponent arrangement is ideal for detecting the light intensity contrast across an edge to serve black-and-white vision (Wiesel and Hubel, 1966). In central vision, the center of the receptive field is comprised of a single L or M cone, with the surround comprised of a mixture of L and M cones. The resulting spectral opponency makes them differentially responsive to wavelengths of light; thus, midget RGCs carry both color and achromatic spatial information. The standard double-duty theory holds that all midget RGCs share the feature of responding to both edge contrast for spatial vision and specific wavelengths for color vision and that the two types of information are disentangled by circuitry in the cortex. Accordingly, the midget RGCs fall into four classes—either ON or OFF cells with either an L or M cone center—all of which are responsible for both color and spatial vision.

The goal is to answer the fundamental question of *how we see*? To answer it, we need to know, at the neuronal level, precisely how percepts of space and color are separated in the visual system. To the extent that the standard double-duty theory proposes that the signals from the midget RGCs carrying both color and spatial information are sent to the brain, “and the brain figures it out,” it is unsatisfying. It fails to answer the question and lacks predictive and explanatory power. As a solution to this inadequacy of the standard double-duty theory, parallel pathways have been proposed originating in the outer retina, with the midget RGCs serving high acuity black-and-white spatial vision and an anatomically and morphologically distinct population of RGCs serving color vision (Rodieck, 1991; Calkins and Sterling, 1999).

There is an evolutionary argument for the primary role of midget RGCs in achromatic spatial vision. Midget RGCs are unique to primates. In Old World primates, including humans with normal color vision, midget RGCs are L/M spectrally opponent. However, in New World (NW) primate species (Jacobs and Neitz, 1987), most individuals have dichromatic color vision. The midget RGCs of dichromats are not L/M opponent and are therefore incapable of carrying red/green hue information. Assuming NW primates resemble an earlier stage of the evolution of human vision, this suggests that midget RGCs originally evolved to mediate black-and-white sensations, which may still be the only function of the L vs. M midget RGCs in the human retina.

**FIGURE 1:**
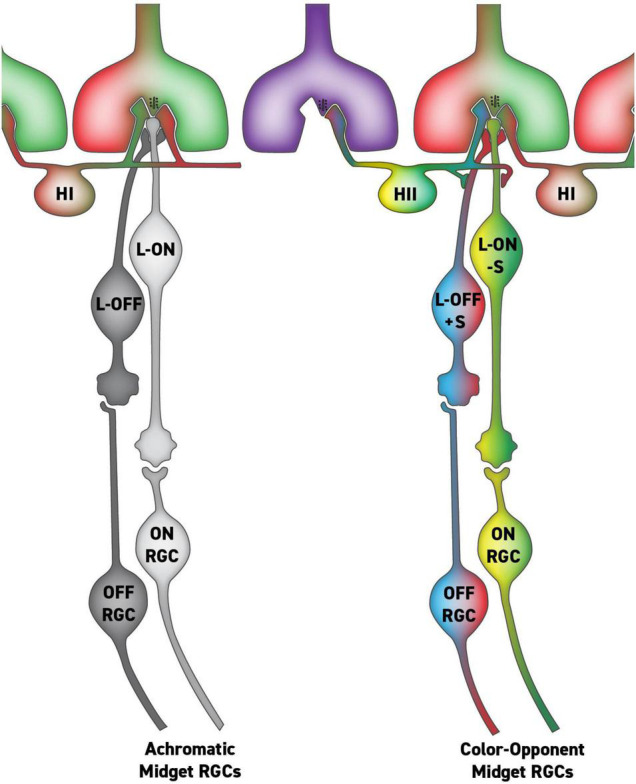
Putative circuit for parallel midget retinal ganglion cell (RGC) pathways with and without S-cone input *via* HII feed-forward signaling. In this circuit, the single-opponent midget RGCs (left) serve achromatic percepts, while the double-opponent RGCs (right) serve hue perception.

The theory of parallel RGC pathways for color and spatial vision was specific and therefore falsifiable. Indeed, as anatomical studies of primate RGCs have gone on, it has become clear that there is no morphologically distinct RGC type that can serve red-green color vision as originally conceived by Rodieck (1991). More recently, we have revised the parallel pathways theory, and instead of there being a morphologically distinct class of RGCs mediating hue sensations, we propose they are mediated by a small subset of midget RGCs with S-cone input that have the specific response characteristics required for color vision (Figure 1). We maintain the idea from the original parallel RGC pathway hypothesis (Rodieck, 1991; Calkins and Sterling, 1999) that most L vs. M midget RGCs lack S-cone inputs and are solely responsible for high acuity black-and-white spatial vision (Puller et al., 2014a; Schmidt et al., 2014, 2016; Neitz and Neitz, 2017). However, morphological studies of the circuitry of the primate retina suggest that a small population of midget RGCs may receive S-cone input via feed-forward from HII horizontal cells conferring the requisite properties to serve our hue perception (de Monasterio, 1979; Tailby et al., 2008; Puller et al., 2014a,b).

Several recent lines of evidence support this unorthodox, parallel pathway theory of midget RGC-mediated black-and-white and color vision. For example, it has recently become possible to stimulate individual cones in humans and ask them what they see (Sabesan et al., 2016). Suppose most midget RGCs only serve black-and-white percepts while a small subset serve hue percepts. In that case, in the central retina most cones should form the receptive field center of achromatic midget RGCs and stimulating them should elicit the achromatic sensation of “white” when probed with a tiny flash of light. At the same time, only a small subset of cones that are in the center of hue encoding neurons should elicit hue sensations of blue, green, red, or yellow. This is a counterintuitive prediction because almost everyone would predict that probing an L cone would elicit the sensation of red and an M cone the sensations of green. Nonetheless, Sabesan and colleagues found precisely the result predicted by the new parallel pathway theory. Most cones elicited sensations of white, but a small subset of cones consistently elicited a hue percept when probed (Sabesan et al., 2016).

The parallel pathways hypothesis was tested in a second experiment that employed adaptive optics imaging (Neitz et al., 2020). Viewing conditions removed chromatic and achromatic optical aberrations, after which a forced-choice paradigm was used to measure contrast sensitivity functions for red-green sinusoidal chromatic gratings under two conditions. The first condition measured contrast thresholds for detecting a red-green grating pattern from uniform, un-patterned distractor targets. In the second condition, the spatial resolution of hue perception was measured for a series of spatial frequencies by requiring subjects to pick out a red-green grating from isochromatic grating distractors that varied only in intensity. Consistent with conventional measures of detection threshold, subjects detected red-green gratings from spatially uniform distracter targets with a high-frequency spatial cutoff of 28–30 c/deg. However, subjects could discriminate colored from isochromatic gratings with a high spatial frequency cutoff of only 10–12 c/deg, an acuity that matches that found for S-cone gratings. Thus, subjects detected the spatial structure of red-green wavelength content at high spatial frequencies without perceiving hue sensations—at high spatial frequencies, the colored gratings were indistinguishable from achromatic ones. Subjects could only perceive the hue of the gratings at much lower spatial frequencies. This is what was predicted by the theory that achromatic and hue sensations are separated at the level of two sub-populations of midget RGCs, one serving high acuity black-and-white vision and a second more sparse mosaic serving hue perception at a lower spatial resolution (Neitz et al., 2020).

The parallel pathways hypothesis may explain recordings from neurons in the primary visual cortex (VI) which receive direct input from the axons in the lateral geniculate nucleus (LGN), which relay signals from midget RGCs in the retina. Most VI neurons respond well to achromatic (dark-light) modulation. In contrast, about 5–10 percent of neurons in V1 respond robustly to purely chromatic modulation but not to achromatic modulation (Solomon and Lennie, 2007). Thus, it appears that the outputs of the midget RGCs are split to form a large population of cortical cells that respond well to black-white edges responsible for high acuity achromatic vision and a second smaller population of color cells that don’t respond to black-and-white at all. S-cone input to a small subset of midget RGCs may explain this. For the proposed small population of midget RGCs that receive S-cone input from HII horizontal cells, the S-cone input is always opposite in sign to L or M cone center (Puller et al., 2014b). An S vs. L or S vs. M cone center will not respond to white light because white contains short, middle, and long wavelengths that stimulate S, M, and L cones equally if the receptive field is normalized white (Neitz et al., 2002). Equal stimulation of S and L cones or S and M cones by white light will cancel the response of S vs. M and S vs. L center midget RGCs. They would be tuned to respond to distinct but overlapping regions of the spectrum to mediate hue sensations, but they would not respond to black or white.

A theory of cortical wiring, originated by Donald Hebb (1949), is often summarized as “Cells that fire together wire together.” M vs. L midget RGCs without S-cone input respond strongly to black-white edges. M-ON and L-ON midget RGCs are excited by white and inhibited by black; being highly correlated in their responses, neurons carrying their signals are likely to be wired together in the cortex, as will neurons carrying midget cell M-OFF and L-OFF signals. So wired, these could correspond to the achromatic neurons in V1. Since they do not respond to black or white, neurons with S-cone inputs carrying hue signals to the cortex would not correlate with each other or the achromatic neurons. Thus, they would not “wire together” but instead form separate classes of cortical neurons responsible for color vision. Accordingly, only the small subset of midget RGCs receiving input from all three cone types would pass hue signals to higher cortical levels, and M vs. L midget RGCs that respond to black and white edges would be responsible for high acuity vision and percepts of black-and-white.

In this investigation, we test the idea that signals from midget RGCs can be separated at the level of the cortex to serve black-and-white and color vision using a computational approach, to determine if the cortex could use unsupervised learning—a type of algorithm that learns patterns from untagged data, the computational equivalent to Hebbian learning—to efficiently separate the outputs of L vs. M midget RGCs based only on the correlation of activity with visual experience over time. Our computations separate ON- and OFF- midget RGC signals as required to serve black-and-white vision: L ON-center and M ON-center midget RGCs are sorted together as a single cluster, as are L OFF-center and M OFF-center, serving white and black percepts, respectively. According to our model, signals from L center and M center midget RGCs are indeed too highly correlated to be separated at a higher level of the visual pathway and thus cannot mediate the red and green hue channels.

Furthermore, our computations shed light on the morphological and physiological properties of RGCs that *would* be capable of mediating hue perception: they must be responsive to rapid changes in spectral reflectance but unresponsive to black-and-white edges. Given these properties, their activity would be decorrelated from the midget RGCs that are particularly sensitive to achromatic edges, allowing them to be categorized as separate hue encoding neurons.

## Materials and Methods

To compare the responses of various midget RGCs in the primate retina, we used MatLab (version R2021b, MathWorks) to simulate physiologically based spatially and spectrally opponent receptive fields. We then developed a simple methodology for quantifying their response to the achromatic and chromatic edges formed by the pixels in an RGB image, and calculated the correlation between their response patterns. In this way, our algorithm reveals which signals the cortex could reliably learn to distinguish based on correlation of activity over time and which would be difficult, if not impossible, to disentangle after leaving the retina.

### Simulate Receptive Fields

**FIGURE 2:**
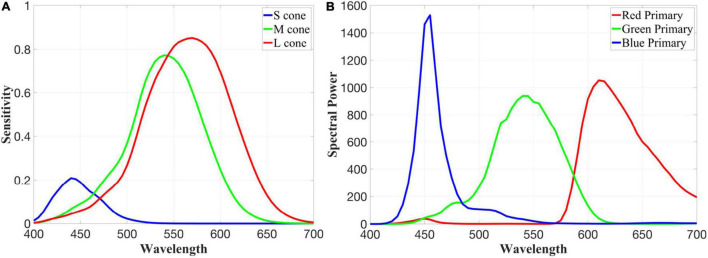
Quantal LMS cone fundamentals **(A)** and the measured red, green, and blue primaries of our LCD display **(B)** that has been adjusted to have a white point equivalent to equal energy white.

Cone spectra are derived using a physiologically based photopigment template (Carroll et al., 2002), with the S, M, and L cones defined by photopigments with spectral peaks at 419, 530, and 559 nm and optical densities of 0.4, 0.22, and 0.35, respectively. After correcting for absorption by the lens and macular pigment (Stockman, 1995-2019), we produce the quantal cone fundamentals pictured in Figure 2A. As a spectral input, we use the spectral power distribution of an LCD display that conforms to the widely used sRGB standard, which we measured using a Konica Minolta CS-2000 (Figure 2B), adjusted to a white point that has the same effect on our cone fundamentals as equal-energy (EE) white, and converted from energy to quanta.

The cone fundamentals are then combined into three single-opponent receptive fields: L-center vs. L+M surround and M-center vs. L+M surround receptive fields created by lateral inhibition from HI horizontal cells, and an S-center vs. L+M surround receptive field created by lateral inhibition from HII horizontal cells. Gain coefficients for the center and surround components of each combination are calculated by using the *lsqnonlin* function in MatLab to ensure that all three single-opponent receptive fields null to the white point of our display.

This produces six gain-adjusted receptive fields: L ON-Center, M ON-Center, L OFF-Center, M OFF-Center, S ON-Center and S OFF-Center. For simplicity, the surround for each of these receptive fields is calculated using an L:M ratio of 2:1, although the program can be tuned to simulate any given L:M ratio. Furthermore, changing the L:M ratio of our model has no impact on the correlations between the L-center and M-center receptive fields, because adjusting the L:M ratio simply changes the relative scaling of the single-opponent receptive fields.

### Calculate Responses to the Edges in an Image

Each receptive field’s response to individual colors is calculated by independently convolving the gain-adjusted response of the center and surround components with the quantal output of the red, green, and blue primaries of our display (see Figure 2). The result of this calculation can now be used to simulate the response to any other color by summing the response to red, green, and blue primaries of our display in proportion to the RGB values of any given pixel in a digital image.

For example, the response of the center of our L vs. (L+M) receptive field to a pixel that is 50% gray is quantified as 50% of the gain-adjusted L-center response to pure red, plus 50% of its response to pure green, plus 50% of its response to pure green.

This takes care of the response to a single light or color. To determine each receptive field’s response to an edge formed by two different lights, we use the equation below:


$$\begin{array}{c} R=\left(L_{1}*C-L_{1}*\frac{2}{3}\,S-L_{2}*\frac{1}{3}\,S\right)-\left(L_{2}*C-L_{2}*\frac{2}{3}\,S-L_{1}*\frac{1}{3}\,S\right),\#\,\left(1\right) \end{array}$$

where L_1_ and L_2_ are the two lights, C represents the gain-adjusted response of the receptive field Center, and S represents the gain-adjusted response of the L+M Surround.

**FIGURE 3:**
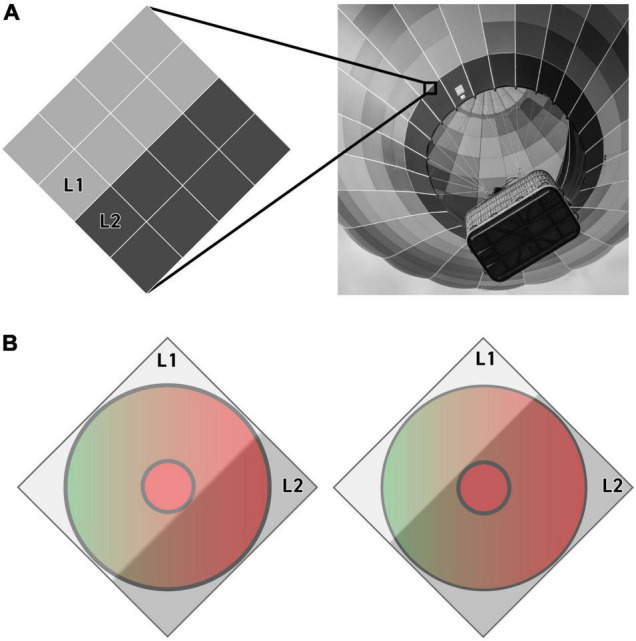
An illustration of the pixels **(A)** and methodology **(B)** that our algorithm uses to quantify a receptive field’s response to an achromatic edge. A light gray (L1) and dark gray (L2) pixel are illustrated here moving across the center and surround of a putative L-center vs. L+M surround receptive field. The receptive field’s response to this particular edge is quantified as the difference between the response to each of the two states shown in B (see Eq. 1).

The response of each individual receptive field to an edge is thus understood as the difference between two states: one in which L1 covers the Center and two-thirds of the Surround while L2 covers only one-third of the Surround, and a second state in which L1 covers only one-third of the Surround and L2 covers the Center and two-thirds of the Surround. We assume that these two states are refreshed constantly as micro-saccades move the edge over each midget ganglion cell receptive field, generating a continuous signal that is equivalent to the difference between these two states (Figure 3).

Note that our model does not consider the spatial orientation of the edge, which could theoretically alter the L:M ratio of the surround response on any given receptive field because of the random distribution of L and M cones in the retina. For simplicity, we treat the surround as one entity that is based on an average L:M ratio of 2:1.

### Image Presentation

**FIGURE 4:**
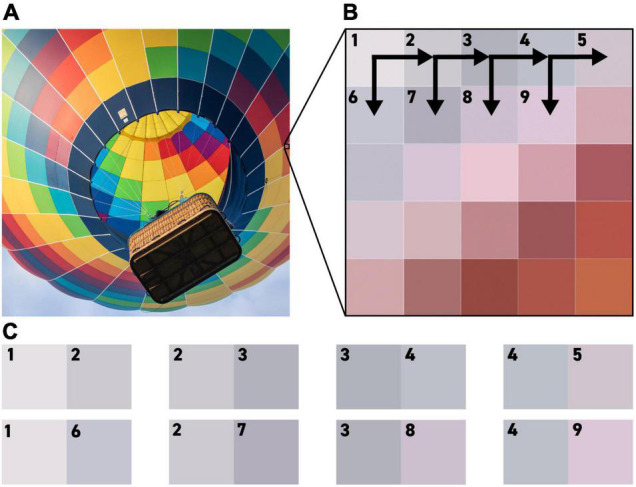
An illustration of the methodology by which pixels from an RGB (Red, Green Blue) image are used to generate testable edges for our algorithm. Each pixel in the test image **(A)** is compared to the pixel to its right and the pixel below it, as seen in **(B)**, to generate the edges shown in **(C)**.

To generate a realistic complement of edges to present to these receptive fields, our model takes as its input any digital RGB image, with each pixel in the image providing up to four testable edges and no pixel used more than once. Combinations where the two pixels are identical are ignored, and each testable edge is shown to each receptive field in turn (Figure 4). Responses to each edge are quantified using the methodology described in the section titled ‘Calculate Responses to the Edges in an Image.’

Because the responses to pairs of identical pixels are ignored, the number of testable edges varies from image to image, but all of the images used still produced millions of data points. For example, the 2000 × 2000-pixel image shown in Figure 4 contains approximately 8 million pixel-to-pixel edges, but even once pairs of identical colors are thrown out, it still contains a total of 5,259,960 testable edges that we can present to our algorithm.

**FIGURE 5:**
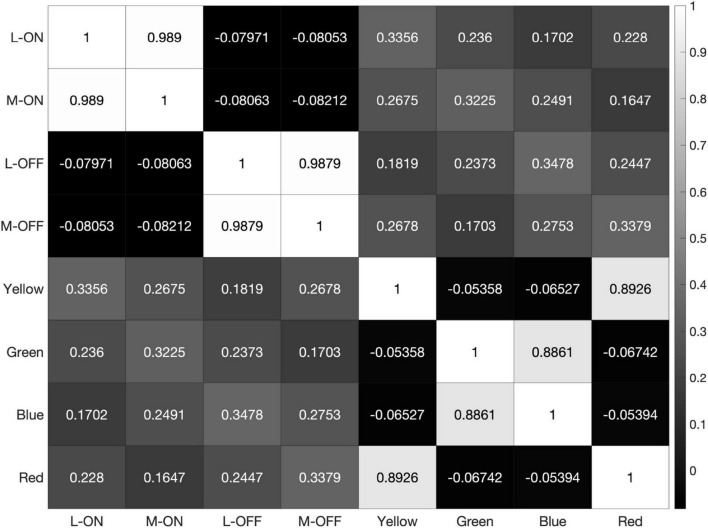
Heatmap of correlations between each of the 8 receptive fields used in our model when exposed to the 2000 × 2000-pixel RGB image shown in Figure 4A. Note the high correlation between the L-ON and M-ON, as well as the L-OFF and M-OFF, receptive fields in rows and columns 1, 2, 3, and 4.

After image presentation, the responses from each receptive field are half-wave rectified and compared to every other receptive field’s response using the *corrcoef* function in MatLab, resulting in a correlation matrix that is visualized as a black-and-white heatmap, as seen in Figure 5. In addition to L-ON, M-ON, L-OFF, and M-OFF receptive fields shown in rows 1, 2, 3, and 4, we are also testing four “double-opponent” receptive fields formed by combining L ON-center and M-ON-center receptive fields with S OFF-center to form Yellow and Green channels, and combining L OFF-center and M OFF-center receptive fields with S ON-center to form Blue and Red channels. These double-opponent receptive fields represent putative color detectors, as they will respond to chromatic edges but remain silent when presented with a black-and-white edge.

The heatmaps produced by our model reveal the correlation, or lack thereof, between all 8 of these receptive fields, which we have labelled L-ON, L-OFF, M-ON, M-OFF, Red, Green, Blue, and Yellow (Figure 5).

The MatLab live code for our algorithm, all dependencies and a folder containing the images tested in this paper is available on the first author’s GitHub.

## Results

We used our model to simulate the response to a variety of digital images, ranging from photographs of natural scenes to vibrant cartoon characters, grayscale images, and a simulation of Tritan color vision deficiency. Our primary goal was to analyze the response properties of the L-ON, L-OFF, M-ON, and M-OFF receptive fields and determine what percepts could reasonably be attributed to these midget RGCs. L vs. M midget RGCs in trichromatic primates carry both spatially and spectrally opponent stimuli, but these stimuli are only useful if they can be disentangled into separate percepts later in the visual stream. If the L-ON and M-ON responses are highly correlated, it’s unlikely these signals could reliably encode red/green vision, supporting our hypothesis that L vs. M midget RGCs without S-cone input exclusively mediate achromatic, black-and-white vision through the OFF- and ON- pathways, respectively.

**FIGURE 6:**
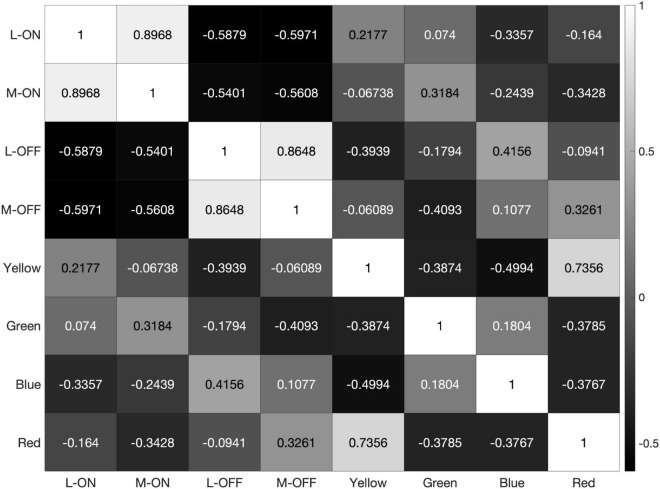
Heatmap of correlations between the 8 receptive fields used in our model, when the model is exposed to the 21 possible edges created by combining the primary colors red, green, blue, yellow, 50% gray, white, and black.

When presented with the 21 possible edges formed by the colors red, green, blue, yellow, white, 50% gray and black, the model reveals a correlation of 90% between the L-ON and M-ON receptive fields and 86% between the L-OFF and M-OFF receptive fields (Figure 6). Even in this highly simplified case based on only 21 stimuli, most of which are colored edges, the response correlation between the L vs. M receptive fields are already quite strong.

**FIGURE 7:**
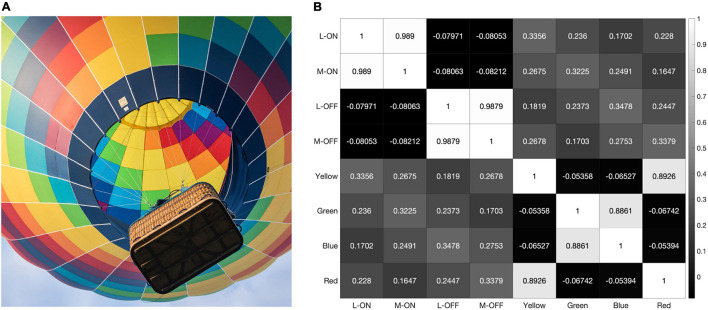
When presented with the image shown in panel **(A)** our model produces the correlation matrix in panel **(B)**.

As the model is presented with more data, the correlations between the L vs. M receptive fields trend towards 100%. When presented with the 2000 × 2000-pixel image from Figure 4, which provides over 5 million testable edges, the correlations between the L-ON and M-ON, as well as the L-OFF and M-OFF, receptive fields jump to 99% (Figure 7).

**FIGURE 8:**
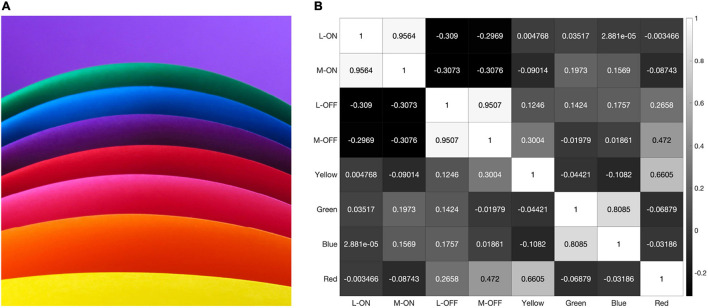
When presented with the illustration shown in panel **(A)** our model produces the correlation matrix in panel **(B)**.

The response pattern is similar when the model is presented with the saturated illustration shown in Figure 8. This illustration was selected because it contains each of the primary colors and very few pure black-and-white edges. Black-and-white edges would be expected to provide the strongest decorrelative effect between the single-opponent and double-opponent receptive fields in our model and a strong correlative effect on the responses of the two single-opponent receptive fields. This is because the double-opponent color detectors do not respond to a black-and-white edge, while both L vs. M receptive fields generate similar responses to this stimulus.

However, the model still reveals a high correlation of over 95% between the L-ON/M-ON and L-OFF/M-OFF receptive field responses (Figure 8).

**FIGURE 9:**
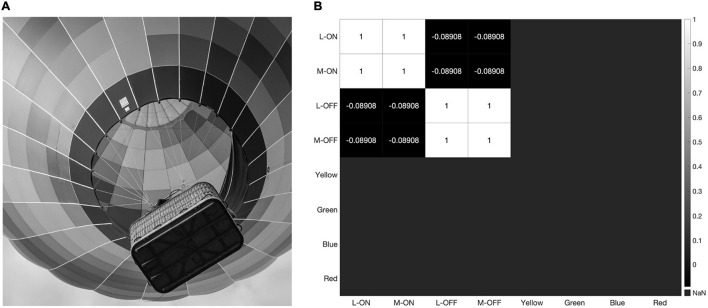
When presented with a grayscale image **(A)** our model produces the correlation matrix in panel **(B)** where the L-ON/M-ON and the L-OFF/M-OFF receptive fields are perfectly correlated with each other, while the putative color-detecting ganglion cells in our model do not respond at all.

The strong impact of black-and-white edges is further illustrated in Figure 9, where we present our model with a grayscale version of the image used in Figures 4 and 7. The double-opponent receptive fields produced by subtracting the response of the S vs. L/M receptive field from the L vs. L/M and M vs. L/M receptive fields to create putative color detectors no longer respond at all, while the L-ON/M-ON and L-OFF/M-OFF receptive fields are perfectly correlated (Figure 9).

**FIGURE 10:**
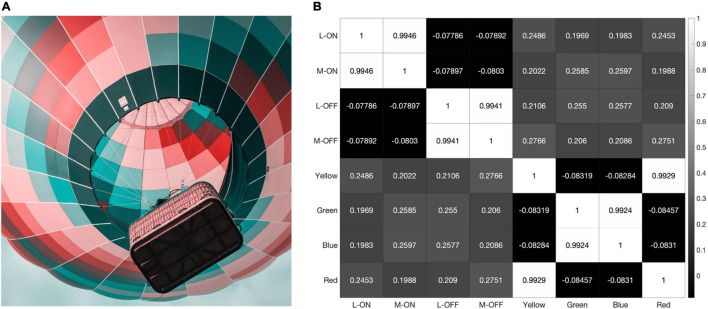
When presented with a simulation of Trian color vision deficiency **(A)** the L-ON/M-ON and L-OFF/M-OFF receptive fields in the resulting correlation matrix **(B)** are still highly correlated.

Finally, we converted the same hot air balloon photograph from Figures 4 and 7 into a simulation of Tritan color vision deficiency using a process called LMS Daltonization (Viénot et al., 1999) that compresses the RGB values along the L vs. M axis, eliminating the S cone contribution (Figure 10). Even in a Tritanopic world where all the colors in our RGB image sit on the L vs. M axis in color space, the correlation between L-ON and M-ON receptive fields is over 99%. It appears that the signals emerging from pure L vs. M opponency via the midget-parvocellular pathway, even in this extreme example, are far more similar than they are different.

## Discussion

DeValois et al. (1966) were the first to systematically and quantitatively compare single cell physiological data with data from experiments on color perception. Based on this, they proposed a theory to account for the subcortical processing of color and luminance information. The three types of LGN cells they described were later identified as receiving input from three distinct morphological types of RGC: midget, small bistratified and parasol cells that have subsequently been associated with red-green, blue-yellow, and achromatic percepts. The idea that these three types of RGCs serve three perceptual dimensions is attractive and versions of the theory where L vs. M, S vs. L/M and non-opponent neurons serve percepts of red/green, blue/yellow and black/white, respectively, still dominate popular science accounts of how we see. However, here we have reviewed and presented evidence that two of the main tenants of the theory are wrong. (1) Parasol RGCs are too sparsely distributed to serve high acuity black-and-white vision (Masri et al., 2020), and (2) The majority of midget RGCs mediate percepts of black-and-white not red and green hues (Sabesan et al., 2016). The paper of DeValois et al. (1966) was named a “citation classic” by the publication *Current Contents* in 1981 (DeValois, 1981). In this review of his seminal work, DeValois commented “I would not now, 15 years later, want to bet very much on the validity of the model we put forth. However, as has often been noted, a theory is historically overthrown not by contrary data but only by a better, more comprehensive theory; since neither we nor others have yet presented one, our original paper still tends to be widely quoted” (DeValois, 1981).

Theories are the heart of science because they generate predictions that can be tested by experiment. DeValois pointed out in 1981 (DeValois, 1981) that experimental observations had contradicted the theory, particularly with regard to the fact that “[color] opponent cells also carry brightness information.” Since then, a great deal of additional data has accumulated indicating that L/M opponent cells serve black-and-white percepts. “A better, more comprehensive theory” of how we see the hues of red, green, blue, yellow and black-and-white—one that would satisfy most vision scientists and overthrow orthodox ideas—needs more research effort. However, here we have focused on making progress in understanding how we see black-and-white.

There are, of course, limitations to the analysis presented here. By calculating responses using only sRGB stimuli, we limit the variety of potential cone responses to those that can be produced using only a combination of the three primaries of a display. This may have an impact on the correlations between the hue encoding receptive fields used in our model, because the “Red vs. Green” mechanism presented here produces significant responses to stimuli that fall below the peak of our display’s blue primary. However, for the purposes of this investigation, where our primary concern was the response of single-opponent L vs. M receptive fields, we believe that using a standard sRGB input has had little impact on the conclusions presented herein.

As we introduced above, the idea that midget RGCs do double duty is reasonably well accepted by vision scientists. What’s controversial is the idea that the majority of midget RGCs serve only black-and-white percepts. Virtually every midget RGC carries both spatial and color information. The proposal that the activity of most midget RGCs is associated with only black/white percepts in the cortex has been interpreted to mean that the visual system is “throwing away” the hue information. This doesn’t sit well with those who believe that the visual system is an efficient encoder optimized to transmit information.

The results of the experiment, introduced earlier, where contrast sensitivity was measured under viewing conditions in which chromatic and achromatic optical aberrations were removed, demonstrates that color information is not thrown away (Neitz et al., 2020). Our visual system uses color information for several different purposes. It can use color information to segment objects for form vision under conditions where light intensity information is a weak or nonexistent cue but there are differences in spectral reflectance. Subjects were able to “see” a purely colored red and green grating at very high spatial frequencies up to 28–30 c/deg. However, the associated percept was achromatic, not red and green. A second use of color vison is to gain information about the surface reflectance of objects. This is encoded in the form of hue information by our visual system. We use information about surface reflectance to identify objects and learn about their internal contents. For example, the hue instantly tells us whether an object is a lemon or a lime. It also tells us about the internal state of an object, allowing us to differentiate a ripe banana from an unripe one. However, surface reflectance information is available at very low spatial frequencies so extracting it only requires a very sparce set of hue detectors. Thus, two parallel sets of midget RGCs represent a very efficient use of color information: one composed of a fine mosaic that can use both wavelength and intensity information for high acuity spatial vision, and another lower spatial resolution mosaic capable of using spectral information to inform us about an object’s internal contents and identity.

Each midget RGC carries an increment of information about color and an increment about the distribution of light across an edge. However, no single midget RGC carries sufficient information to differentiate color from an edge. Previous double-duty models proposed that midget RGCs with different receptive field properties containing different pieces of information were linearly combined in the cortex to extract color and spatial information (Derrico and Buchsbaum, 1991; DeValois and DeValois, 1993). For example, in the Derrico and Buchsbaum model (Derrico and Buchsbaum, 1991) signals from L-ON-center and M-ON-center cells are added to form the achromatic channel and they are subtracted to form the red-green hue channel. However, it was never explained how the cortex would be able to identify neurons with L vs. M cone centers or how it would *“know”* to add or subtract them to extract color and spatial information. Here we propose a way to solve this problem. It is well accepted that signals from LGN neurons converge additively on cortical cells (Hubel and Wiesel, 1962) and that Hebbian mechanisms can explain how cortical cells are wired (Hubel, 1988). In our model, we demonstrate that the activity of L-ON-center cells and M-ON-center cells are highly correlated when exposed to visual scenes, as are L-OFF-center and M-OFF center cells, explaining how achromatic cortical cells as proposed by Derrico and Buchsbaum (1991) would be wired. Such cells would maintain the ability to respond to edges defined by intensity or wavelength differences, so the wavelength information is not lost with regard to its usefulness in segregating objects, but they would not be used to encode the perception of hue.

Instead of subtracting L-ON-center and M-ON-center cells to produce cortical cells that encode hue as proposed in earlier double-duty models (Derrico and Buchsbaum, 1991; DeValois and DeValois, 1993), we propose an alternative solution that is more parsimonious and has greater explanatory power. We propose the retina contains a small population of hue-specific midget RGCs which are wired to respond to hue but not black-and-white as the result of known S-cone feedforward circuitry in the outer retina (Puller et al., 2014a,b). This explains why circuits for the four different hue percepts—red, green, blue and yellow—all have S-cone inputs (Mollon and Jordan, 1997; Stockman and Brainard, 2010; Schmidt et al., 2014, 2016; Neitz and Neitz, 2017). The fact that they do not respond to black-and-white edges decorrelates their responses from the L/M midget signals, separating hue and spatial information in the cortex by the same Hebbian mechanism that combines midget signals to form the achromatic cortical neurons.

Müller’s law of specific nerve energies is a fundamental principle in neuroscience (Müller, 1833). The idea is that a neuron will always be associated with the same percept no matter the nature of the stimulus that caused it to respond. We argue that most cortical cells sum ON signals or OFF signals and are associated with percepts of white and black, respectively, when they are simulated by spatial patterns of wavelength or intensity.

The computational approach presented here bears out the predictions of this alternative parallel pathway theory of edge detection and color appearance. It illustrates the difficulty of finding any stimuli that do not produce highly correlated responses between L-ON and M-ON, as well as L-OFF and M-OFF, midget RGC receptive fields, while simultaneously showing how the responses of the ON and OFF cells are consistently decorrelated from one another, allowing for easy distinction between the lighter and darker sides of a high-frequency edge. As such, the parvocellular pathway can be conceptualized as an artist that makes use of two distinct yet equally important tools: a fine-tipped pen for creating a precise line drawing of the image falling on the fovea, and a set of four broad-tipped markers for filling in the colors.

## Data Availability Statement

The raw data supporting the conclusions of this article will be made available by the authors, without undue reservation.

## Author Contributions

JN and DR conceived the project. DR wrote an initial draft of the manuscript and created the MatLab model used in this investigation. All authors edited the final version of the manuscript.

## Conflict of Interest

The authors declare that the research was conducted in the absence of any commercial or financial relationships that could be construed as a potential conflict of interest.

## Publisher’s Note

All claims expressed in this article are solely those of the authors and do not necessarily represent those of their affiliated organizations, or those of the publisher, the editors and the reviewers. Any product that may be evaluated in this article, or claim that may be made by its manufacturer, is not guaranteed or endorsed by the publisher.
